# Targeting Estrogen Receptor Sites in Human Breast Cancer Cell Line T47D With Copper Conjugates of Nonsteriodal Anti-inflammatory Drug Derivatives: Antiproliferative Activity of Ketoprofen Derivative and its Copper Complex

**DOI:** 10.1155/MBD.2001.73

**Published:** 2001

**Authors:** Dilip Kumar Saha, Shreelekha Padhye, Subhash Padhye

**Affiliations:** 1 Department of Chemistry University of Pune Pune 411007 India

## Abstract

A square planar copper complex of derivatized NSAID drug (Ketoprofen thiosemicarbazone [3-benzoyl-α-methyl benzene acetic acid thiosemicarbazone]), is characterized by elemental analysis, spectroscopy, electrochemistry and magnetic susceptibility studies which exhibits dose-dependent and enhanced antiproliferative effects on human breast cancer cell line T47D rich in progesterone receptors.


### TARGETING ESTROGEN RECEPTOR SITES IN HUMAN BREAST CANCER CELL LINE T47D WITH COPPER CONJUGATES OF NONSTERIODAL ANTI-INFLAMMATORY DRUG DERIVATIVES: ANTIPROLIFERATIVE ACTIVITY OF KETOPROFEN DERIVATIVE AND ITS COPPER COMPLEX

Dilip Kumar Saha, Shreelekha Padhye, and Subhash Padhye*

Department of Chemistry, University of Pune, Pune-411007 India

<sbpadhye@chem.unipune.ernet.in> Fax: +91-20-5653899/5651728

**Abstract**

A square planar copper complex of derivatized NSAID drug (Ketoprofen thiosemicarbazone [3-benzoyl-α-methyl benzene acetic acid thiosemicarbazone]), is characterized by elemental analysis, spectroscopy, electrochemistry and magnetic susceptibility studies which exhibits dose-dependent and enhanced antiproliferative effects on human breast cancer cell line T47D rich in progesterone receptors.

### Introduction

The pharmacology of nonsteroidal anti-inflammatory drugs (NSAIDs) and roles of cyclooxygenase enzymes, viz. COX-1 and COX-2, have now been integrated into a unified model [1,2] suggesting that inhibition of both COX isoforms by NSAIDs leads to prevention of cancers in animal model through induction of apoptosis and stimulation of immune serveillance.[3,4] The actual mechanism of this action, however, is not known.

Ketoprofen (I) is a widely used NSAID in clinical practice possessing a variety of biological activities[5] which has close structural resemblance to non-steroidal antiestrogens viz. Trioxifene (II) and Keoxifene (III).

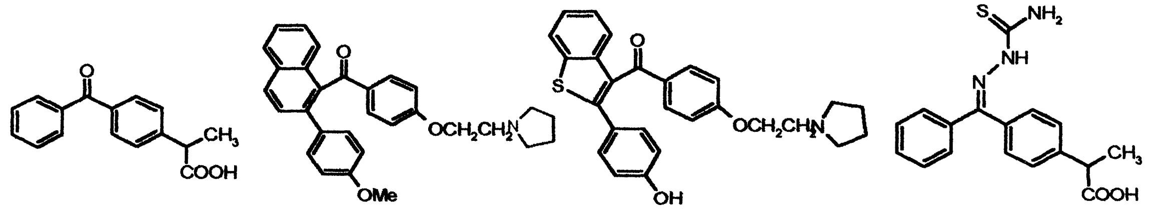

(I) (II) (III) (IV)

Since II and III have been shown to compete with estradiol for estrogen receptor (ER) binding site and thereby prevent the growth of malignancies in breast cancers,[6,7] I may also be looked upon as a potential anticancer agent. In order to exploit ER binding affinities of I for the purposes of evolving compounds which can deliver cytotoxic drugs to the hormone responsive cancer cells we decided to modify ketoprofen moiety with thiosemicarbazone functionality which has been known to attribute antiproliferative properties to several organic compounds of clinical significance.[8-11] Combination of clinically used NSAIDs with cytotoxic drugs has also been shown to exert synergic effects on the cytotoxicities of many anticancer drugs which is of potential clinical significance in the treatment of certain cancers.[12] Additionally appendage by the thiosemicarbazone pharmacophore allows for metal conjugation especially with copper leading to enhancement in selective binding to the estrogen receptors as shown by Predki and Sarkar.[13,14]

In the present communication we describe the synthesis and characterization of ketoprofen thiosemicarbazone (KFTSC, IV) and its Cu (II) conjugate and evaluation of their antiproliferative activities against human breast cancer cells T47D, which is a progesterone receptor rich, and ER sensitive cell line.[15]

**Material and method**

All chemicals used in the syntheses of ligand and copper conjugates were of AR grade while solvents were distilled prior to their use. Ketoprofen (Aldrich), thiosemicarbazide (Sisco Chem. Pvt. Ltd.) and $CuCl_2.2H_2O$ (Qualigens) were used as supplied.

*Synthesis of ligand*

Ketoprofen thiosemicarbazone (**IV**) was prepared by reacting **I** (0.254g, 0.001mole) and thiosemicarbazide (0.091g, 0.001mole) both dissolved in methanol (10 ml) with one drop of concentrated hydrochloric acid and bringing the reaction mixture to a reflux on the water bath for two hours. A white colored microcrystalline product separated out when the mixture was allowed to cool. It was washed with ether and dried in vacuum.

Anal. Calc. For **KFTSC (IV)**: %C, 62.37; %H, 5.23; %N, 12.83. Found: %C, 62.21; %H, 5.12; %N, 12.81. IR (nujol),ν(OH) 3166 cm $^{-1}$, ν(C=N) 1595 cm$^{-1}$, ν(C=S) 924 cm.$^{-1}$

*Synthesis of Copper Complex*

The copper complex was synthesized by refluxing the methanolic solutions of the ligand and copper chloride dihydrate in 1:1 molar ratio for 25 minutes. The green precipitate of the complex was filtered, washed with cold methanol and dried in vacuum.

Anal. Calc. For [ **Cu(KFTSC)Cl$_2$**]: %C, 44.21; %H, 3.71; %N, 9.10; %Cu, 13.76. Found: %C, 44.20; %H, 3.68; %N, 9.11; %Cu, 13.72. IR (nujol); ν(OH) 3167cm$^{-1}$, ν(C=N) 1571 cm$^{-1}$, ν(C=S) 903 cm.$^{-1}$

*Instruments*

Elemental analyses were carried out using HOSLI CHN analyser. Infrared spectra were recorded in KBr discs in the range of 4400-450cm$^{-1}$ on a Perkin-Elmer 1615 FTIR instrument while electronic spectra were recorded on Genesis-2 UV-VIS Spectrophotometer in the range 300-1000 nm. The magnetic susceptibility of the complex was measured at 300K on a Faraday Balance having field strength of 7000 KG using $Hg[Co(SCN)_4]$ as a calibrant. Cyclic Voltammetric measurements were made in DMF solvent on a Bioanalytical System BAS CV-27 with XY-recorder using a platinum disc as working electrode against SCE and platinum wire as the reference and auxiliary electrodes respectively. Tetraethyl ammonium perchlorate (TEAP) was used as a supporting electrolyte.

*Cell Culture*

T47D cell were routinely cultured in Dulbecco's modified Eagle's medium (DMEM) [17] with phenol red, Penicillin (50 U/ml), Streptomycin (50μg/ml) and 10% Foetal calf serum (FCS) (GIBCO, Myoclone). Experiments were conducted on cells seeded into 48 well culture plates at densities over the range of 4000 to 10000 cells per cm.$^2$ Cells were kept at 30°C in an atmosphere of 5% $CO_2$ in air.

*Tritiated thymidine uptake assay*

T47D cells were incubated over a period of 48 hours with varying concentrations of the test compounds in 48 well plates. At 48 hours, [$^3$H] thymidine was added to the cells and incubated at 37°C for 2/3 hours. After removing the medium, cells were precipitated with ice cold 10% Trichloro acetic acid (TCA) and kept at 4°C for 1-2 hours. Finally, the cells were washed with ice cold Phosphate buffered saline (PBS), to remove unbound tritium while DNA was hydrolysed with 0.5M NaOH/0.1% Triton X-100. Contents of each well were mixed with Scintillation fluid and radioactivity was measured in Liquid Scintillation Counter.

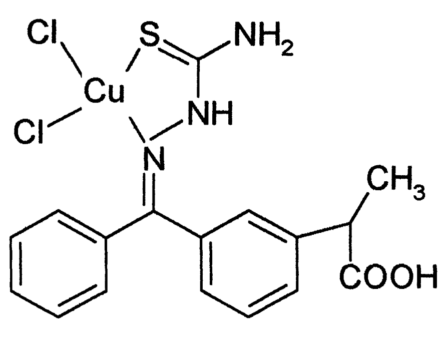

**Figure 1.** Proposed structure of complex [$Cu(KFTSC)Cl_2$]

**Results and Discussion**

The reaction of KFTSC with $CuCl_2 .2H_2O$ in methanol results in a neutral, green complex where metal coordination takes place through thione sulfur and imine nitrogen atom as shown in **Figure 1**.

The IR spectrum of the ligand exhibits a broad band around 3166 $cm^{-1}$ which can be ascribed to the H-bonded OH of the carboxyl group.[18] The strong band at 1697 $cm^{-1}$ is attributed to the C=O stretching band of the carboxylate group while the absorption at 1654 $cm^{-1}$ is assigned to the stretching frequency of 3-benzoyl carbonyl group respectively.[19] Condensation with thiosemicarbazone moiety results in the loss of band at 1654 $cm^{-1}$ confirming successful derivatization of the benzoyl carbonyl group. Two additional bands can be seen at 3417 and 3340 $cm^{-1}$ which are due to asymmetric and symmetric stretches of the amino group.[19] The imine $\nu_{(C=N)}$ and thiocarbonyl $\nu_{(C=S)}$ functionalities are observed at 1595 and 924 $cm^{-1}$ which are shifted to 1571 and 903 $cm^{-1}$ respectively on copper conjugation indicating their involvement in metal coordination.[8, 20]

The electronic spectra of **IV** in DMF shows a band at 31056 $cm^{-1}$ due to extended conjugation in the thiosemicarbazone moiety while its copper conjugate[8] exhibits a moderately intense band at 27027 $cm^{-1}$ due to ligand to metal charge transfer.[8] The broad band observed for the metal-based transition at 17153 $cm^{-1}$ is attributable to a combination of the transitions $^2B_{1g} \rightarrow {}^2E_g$ and $^2B_{1g} \rightarrow {}^2A_{1g}$ respectively in a square planar configuration[21] which is further supported by the observed magnetic moment of 1.90 BM for this complex. The cyclic voltammograms for KFTSC and its copper complex in DMF solvent are shown in **Figure 2.** The CV profile of the copper complex exhibits two successive reduction peaks at -0.68V and -1.28V, none of which show counterparts on the reverse anodic side.

**Figure 2.** Cyclic voltammograms (100mV/s) of a $10^{-3}$M solutions of (A) KFTSC, (B) $[Cu(KFTSC)Cl_2]$ in DMF with inset showing the scan rate dependence (mV/s) $Cu^{2+/1+}$ redox peak centered at +0.40V.

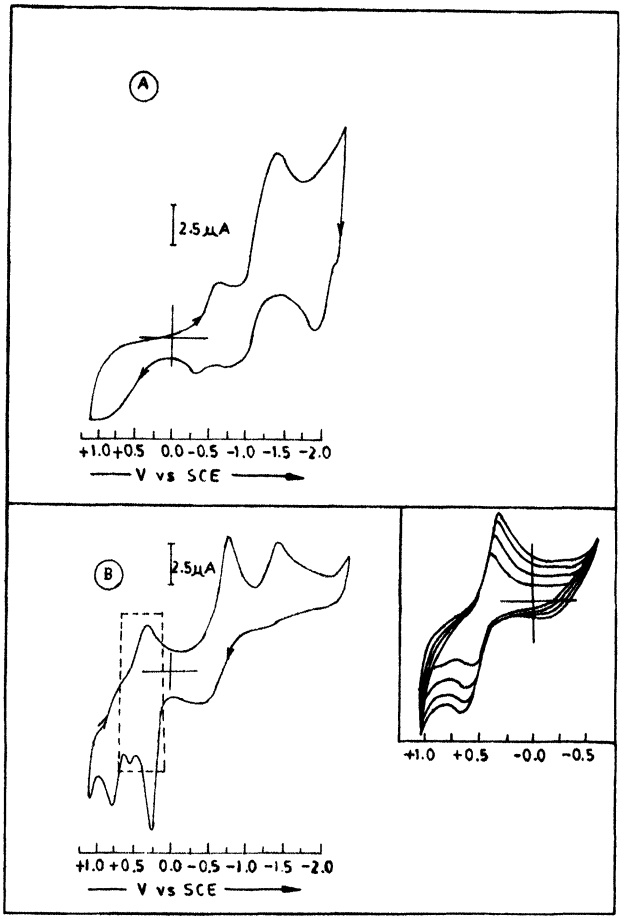

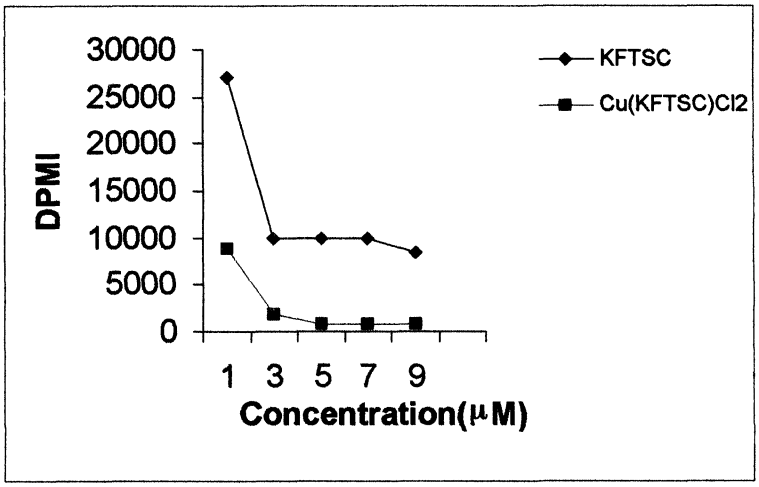

**Figure 3.** Dose-dependent DNA inhibition of KFTSC and [Cu(KFTSC)$Cl_2$] against human breast cancer cell lineT47D.

Treatment of KFTSC and [Cu(KFTSC)$Cl_2$] with the T47D cell line shows inhibition of DNA synthesis in a dose-dependent manner. The $IC_{50}$ value of the ligand (3μM) is lowered on complexation with copper (>1μM) indicating that metal complexation with copper clearly offers an advantage in designing antiproliferative compounds especially against hormone responsive cancers. The selective affinity of copper ions towards the intranuclear estrogen receptor binding sites and consequent inhibition of DNA dimerization noted by Predki and Sarkar.[13] may be contributing to the observed enhancement in antiproliferative activities of these copper conjugates.

**Acknowledgement**

SP would like to thank the British Council for the Visitorship Program. DKS would like to thank ICCR and Govt. of Bangladesh for financial assistance and deputation respectively.

**References**

1. W.L Smith, L. J. Marnett, *Biochem. Biophys.Acta*, **1083**, 1 (1991).

2. C. S. Williams, R. N. DuBois, *Am. J. Physiol.*, **270**, G393 (1996).

3. R. A. Gupta, R. N. DuBois, *Gastroenterology*, **114**, 1095 (1998).

4. S. J. Shiff, B. Regas, *Nature Med.*, **5**, 1348 (1999).

5. S. Kobayashi, S. Okada, H. Yoshida, S. Fujimura, *Tohoku J. of Expt. Med.*, **181**,361 (1997).

6. R.A. Magarian, L.B. Overacre, S. Singh, K. L. Meyer, *Current Med. Chem.*, **1**, 61 (1994).

7. S. Grag, S. Durani, R. S. Kapil, *J. Med. Chem.*, **28**, 492 (1985).8

8. A. Murugkar, S. Padhye, S. Guha-Roy, U. Wagh, *Inorg. Chem. Commun.*, **2**, 545 (1999).

9. S. Padhye, R. Chikate, A. Kumbhar, J. M. Shallom, M. P. Chitnis, *Biometals*, **5**, 67 (1992).

10. R. C. DeConti, B. R. Toftness, K. C. Agarwal, R. Tomchick, J. A. R. Mead, J. R. Bertino, A. C. Sartorilli, W. A. Creasey, *Cancer Res.*, **32**,1455 (1972).

11. I. H. Krakoff, E. Etcubanas, C. Tan, K. Mayer, V. Bethune, J. H. Burchenal, *Cancer Chemother. Rep.*, **58**, 207 (1974).

12. P. Duffy, C. J. Elliott, R. A. Occonor, M. M. Hcenam, S. Coyle, I.M. Cleary, K. Kavanagh, S. Verhacgen, C. M. Oloughlim, R. NicAmhlaoibh, M. Clynes, *European J. Cancer*, **34**, 1250, (1998).

13. P. F. Predki, B. Sarkar, *J. Biol. Chem.*, **267**, 5842 (1992).

14. Y. Maaroyfi, A. Cleeren, G. Leclereq, *J.Biol.Inorg.Chem.*, **3**,508 (1998).

15. S. Kodali, Mburkley, R. N. Koushik, C. Taylor, V. K. Moudgil, *Biochem. Biophys.Res. Commun.*, **202**, 1413 (1994).

16. N. Gokhale, S. Padhye, C. Newton, R. Pritchard, *Metal Based Drugs*, **7**, 121(2000).

17. C. J. Newton, R. Burie, T. Trapp, S. Brockmeier, U. Pagotto, G. K. Stalla, *J. Steroid Biochem. Molec. Bio.*,**48**, 481 (1994)

18. R. M. Silverstein, G. C. Barslar, T. C. Morrill, " *Spectrometric Identification of Organic Compounds*" 5th Edn., John Wiley, & Sons, Inc., 117 (1991).

19. Ibid, p.123.

20. D. X. West, A. E. Liberta, S. B. Padhye, R. C. Chikate, P. B. Sonawane, A. S. Kumbhar, R. G. Yerande, *Coord. Chem. Rev.*, **123**, 49 (1993).

21. K. M. Ibrahim, M. M. Berheit, *Trans. Met. Chem.*, **13**, 230 (1988).

22. M. E. Bodini, P. E. Bravo, V. Aancibia M., *Polyhedron*, **13**, 497 (1994).

23. V. Eisner, E. K. Eisher, in A. J. Bard, H. Hurd (eds), *Encyclopedia of Electrochemistry of the Elements*, Vol. **XIII**, Marcel Dekker, New York, 349 (1979).

24. A. S. Kumbhar, S. B. Padhye, D. X. West, A. E. Liberta, *Trans. Met.Chem.*, **19**,277 (1994).

25. P. Sonawane, A. Kumbhar, S. Padhye, R. J. Butcher, *Trans. Met. Chem.*, **19**, 277, (1994).

**Received: February 21, 2001 – Accepted: March 14, 2001 –
Accepted in publishable format: March 15, 2001**

